# Psychological Impact of COVID-19 and Lockdown among University Students in Malaysia: Implications and Policy Recommendations

**DOI:** 10.3390/ijerph17176206

**Published:** 2020-08-27

**Authors:** Sheela Sundarasen, Karuthan Chinna, Kamilah Kamaludin, Mohammad Nurunnabi, Gul Mohammad Baloch, Heba Bakr Khoshaim, Syed Far Abid Hossain, Areej Sukayt

**Affiliations:** 1 Department of Accounting, Prince Sultan University, P.O. Box 66833, Riyadh 11586, Saudi Arabia; ssundarasen@psu.edu.sa (S.S.); kkamaludin@psu.edu.sa (K.K.); mnurunnabi@psu.edu.sa (M.N.); asukayt@psu.edu.sa (A.S.); 2 School of Medicine, Faculty of Health and Medical Sciences, Taylor’s University, No. 1, Jalan Taylors, Subang Jaya 47500, Selangor, Malaysia; drgulbuledi@gmail.com; 3 Deanship of Educational Services, Prince Sultan University, P.O. Box 66833, Riyadh 11586, Saudi Arabia; hkhoshaim@psu.edu.sa; 4 College of Business Administration, International University of Business Agriculture and Technology (IUBAT), 4 Embankment Drive Road, Sector-10, Uttara Model Town, Dhaka 1230, Bangladesh; sfa_hossain@yahoo.com

**Keywords:** COVID-19, anxiety, social psychology, university students, Zung’s self-rating anxiety scale (SAS), Malaysia

## Abstract

The COVID-19 pandemic and the lockdown has taken the world by storm. This study examines its impact on the anxiety level of university students in Malaysia during the peak of the crisis and the pertinent characteristics affecting their anxiety. A cross-sectional online survey, using Zung’s self-rating anxiety questionnaire was conducted during the COVID-19 pandemic and lockdown. Out of the 983 respondents, 20.4%, 6.6%, and 2.8% experienced minimal to moderate, marked to severe, and most extreme levels of anxiety. Female gender (OR = 21.456, 95% CI = 1.061, 1.998, *p* = 0.020), age below 18 years (OR = 4.147, 95% CI = 1.331, 12.918, *p* = 0.014), age 19 to 25 (OR = 3.398, 95% CI = 1.431, 8.066, *p* = 0.006), pre-university level of education (OR = 2.882, 95% CI = 1.212, 6.854, *p* = 0.017), management studies (OR = 2.278, 95% CI = 1.526, 3.399, *p* < 0.001), and staying alone (OR = 2.208, 95% CI = 1.127, 4.325, *p* = 0.021) were significantly associated with higher levels of anxiety. The main stressors include financial constraints, remote online teaching and uncertainty about the future with regard to academics and career. Stressors are predominantly financial constraints, remote online learning, and uncertainty related to their academic performance, and future career prospects.

## 1. Introduction

“The impact of the pandemic on people’s mental health is already extremely concerning. Social isolation, fear of contagion, and loss of family members is compounded by the distress caused by loss of income and often employment.”

Dr. Tedros Adhanom Ghebreyesus

Director-General, World Health Organization (2020)

The outbreak of coronavirus disease 2019 (COVID-19), which started in China in December 2019, is a catastrophic calamity that has spread across the entire world at the speed of light. Public health measures have been implemented in almost every country to contain the disease’s transmission [1]. The Centers for Disease Control (CDC) advocates that it is critical to recognize stress symptomsresulting from the lockdowns and the disease itself. During outbreaks of transmittable diseases such as severe acute respiratory syndrome (SARS) [2,3,4] and equine influenza [5], damaging psychological implications have been documented [6,7]. It is apparent that the unswerving psychological and social impacts of the pandemic are inescapable, and it is critical to take steps in building resilience and coping with such damaging consequences of a pandemic [8]. As suggested by [9], it is a timely call for studies investigating the impact of COVID-19 on students’ mental health and the need for immediate interventions.

According to the United Nation’s Educational, Scientific, and Cultural Organization (UNESCO), the pandemic has interrupted the learning of more than one billion students in 129 countries around the world [10]. Many universities worldwide have moved to emergency remote teaching (ERT) via online platforms, further inducing anxiety among the students. Studies on the effect of COVID-19 and lockdowns on college students in China reported significant adverse effects on the students’ psychological well-being and high levels of anxiety [11,12,13]. To date, several studies have been conducted on the effects of the COVID-19 pandemic and lockdowns from the public health perspective. Most of the research has been conducted in China and Western countries, mainly among the general population, healthcare workers, and medical students [12,13,14,15,16,17,18,19]. No such studies have been conducted in Malaysia, yet. Thus, this study hopes to extend the existing literature by empirically evaluating the impacts of the COVID-19 pandemic and the subsequent lockdowns on the socio-psychological well-being and anxiety among Malaysian university students, during the heights of the pandemic and lockdown, between April and May 2020.

We hope the findings of this study could assist colleges and universities in Malaysia in forming a theoretical basis for determining psychological well-being and identifying evidenced-based psychological intervention practices to assist the students in times of similar pandemics in the future. It should also provide guidelines for policymakers on possible mechanisms to moderate the impacts of anxiety on students during such health crises.

In Malaysia, COVID-19 was first detected in January 2020. Cases recorded then were rather scarce and mainly limited to tourists. Local outbreaks started to appear in March 2020; the main cluster was connected to a spiritual gathering, Tablighi Jamaat, in late February and early March, culminating in a sudden and sharp upsurge in local cases and those exported to neighboring countries. Within weeks, Malaysia had registered the highest increasing number of COVID-19 contagion in Asia. Actions to mitigate the pandemic were promptly taken by the Malaysian government. On 18 March 2020, a nationwide “Movement Control Order” (MCO; i.e., lockdown) was declared, aimed at controlling the viral outbreak. Quarantining of infected people and social distancing were implemented to restrict the virus’ spread and reduce interactions with individuals infected with COVID-19. With the MCO, the Malaysian population was confined to a long period of social isolation. The unprecedented rules had significantly altered the lifestyles and social relationships between the people and had presumably developed deep levels of anxiety along with the fear of contracting the infection. Although measures taken primarily reduced the outbreak of COVID-19 in Malaysia, measures such as lockdowns, strict isolation, social distancing, emergency remote teachings, and uncertainty and delays in commencement of schools, colleges, and universities have significant implications on students’ socio-psychological well-being and anxiety levels.

Lazarus et al. [20] (p. 19) defined stress as “a particular relationship between the person and the environment that the person considers to be taxing or exceeding his or her resources and putting his or her well-being at risk.” These authors further postulated that stress is best viewed as an interpretative paradigm between the stressors and the individual’s psychological reactions. Anxiety, being a subcategory of psychological impacts, has attracted less attention, although it is as widespread and conceivably as crippling as depression [21]. Anxiety can be fueled by uncertainty and by fears of risk of harm to self or others. Anxiety is still undiagnosed and under-treated in the global context [22]. In addition to intense feelings of fear or panic [23], anxiety patients may also experience other physiological symptoms, such as feeling weak, fainting, pain or nausea, shivers, rapid breathing, etc. [24]. Anxiety impairs focus and concentration [25], memory [26], and visual motor skills [27].

Specific to this study, early literature has documented the negative influence of pandemics on students’ psychological well-being [28], which has led to acute depression and anxiety [29]. Cao et al. [11] investigated the psychological impact on university students in China during the COVID-19 pandemic. Out of 7143 students studied, 0.9% had severe anxiety, 2.7% had moderate anxiety, and 21.3% had mild anxiety. The study by [12] on 1210 students from 194 cities in China, found 53.8% of respondents having severe to moderate psychological impact, with female students being associated with greater psychological impacts. As suggested in previous studies, refs. [12,30,31,32] also opined that heightened uncertainty and its bearings on students’ academic progress could influence students’ psychological well-being.

Odriozola-González et al. [33] studied the psychological well-being of Spanish university students during the COVID-19 pandemic. The study was conducted from March 28 to 4 April 2020, a fortnight after the lockdown in Spain due to the COVID-19 pandemic. In the sample, 34.19% of respondents showed moderate to extremely severe depression symptoms; 21.34% showed extremely severe anxiety symptoms; and 28.14% exhibited moderate to extremely severe stress symptoms. The incidence of anxiety was higher among students compared to that among the general population. Several stressors have been identified as key factors affecting the students’ anxiety and psychological well-being: a parent or associate being infected by COVID-19; monetary issues and their effects on daily life; educational disruptions [11], effects of the disease on education and potential jobs [12,34]; and sensational broadcasts and inaccurate news reports [35]. Other stressors include students’ place of residence, family income stability [3,36], parents’ psychological status [37], reduced social interactions [38], increased number of new cases and affected provinces [13], and the imposition of travel bans affecting daily life [39].

Lockdowns or quarantines are necessary as protective measures for physical health [8], but prolonged impositions can be detrimental. It is a hostile experience that can cause severe financial stress [40,41] due to loss of employment; social disorders such as social withdrawal, cyberbullying, alcohol misuse, and addiction; and mental health issues such as suicide attempts and depression [42,43]. Even during the SARS outbreak, [44] stated that quarantine was linked to high rates of depression (31.2%) and anxiety (28.9%). Similarly, high anxiety was detected throughout the 2009 H1N1 pandemic [45,46]; a study from China, found that those in quarantine experience monotony, aloneness, irritation, worsening anxiety, and mental distress. These authors further added that COVID-19 has been repetitively labeled a killer virus, mainly on social media (e.g., WeChat), which has prolonged feelings of perceived threats and uncertainty. Compulsory 14-day quarantines and tracking as part of the public health protocols during the pandemic further increase people’s anxiety based on the effects of infection and stigma. Lockdown stressors include separation from family and friends, loss of independence, doubts about the virus’s spread, lockdown length, resentment, monotonous lifestyle, potential scarcity of essential goods, lack of accurate information, monetary loss, and stigma.

The main objective of this study is to determine the level of anxiety among university students in Malaysia during COVID-19 and the MCO period and to determine the associated demographic characteristics. We also want to identify potential stressors among the students during this testing time.

## 2. Materials and Methods

### 2.1. Study Population

To evaluate the level of anxiety among university students throughout the height of COVID-19 and the MCO period in Malaysia, an exploratory study using a cross-sectional online survey was conducted. The study period was between 20 April and 24 May 2020. To ensure a well-spread pool of respondents, the participants were sampled from both private and public colleges and universities from all states in Malaysia. A survey invitation through Google Forms was sent to students via WhatsApp messages, with periodic reminders. Participation in the survey was voluntary and the students’ consents were obtained prior to the start of the survey. The participants were assured regarding the confidentiality of their responses. The research instruments used in this study included basic demographics; gender, age, name of institution, field of study, level of study, year of study, nationality, ethnicity, current mode of study (virtual or online), and students’ living conditions.

### 2.2. Study Instrument

In this survey, anxiety level was assessed using Zung’s self-rating anxiety scale (SAS) self-rating anxiety questionnaire. This instrument was developed based on affective symptoms according to diagnostic criteria and not based on factor analytics studies [47]. Since then, it has been translated and used in many countries. In a recent study [48], Zung’s SAS has demonstrated good psychometric properties (Cronbach’s alpha = 0.897 and intraclass correlation = 0.913). In this study, the English version of Zung’s SAS was used. This instrument employs a Likert-type scale of 1–4; “1 = Never or very rare,” “2 = Sometimes,” “3 = Often,” and “4 = Very Often or always.” Questions 1–5 characterize the emotional pointers of anxiety, whereas questions 6–20 signify the physical symptoms of anxiety. In this study, no differentiation was made between emotional and physical symptoms. For each respondent, the sum of the scores in the 20 items ranges from 20 to 80. The sum of scores are then converted to an “Anxiety Index” with values ranging from 25 to 100. According to Zung [47], an Anxiety Index <45 indicates “Anxiety within normal range,’’ a value in the range of 45–59 indicates “Mild to moderate anxiety,” a value in the range of 60–74 indicates “marked to severe anxiety,” and values ≥75 indicates “Most extreme anxiety.’ In the Google form there was an open-ended question were the students were asked to write their main concerns and worries in this testing time.

### 2.3. Data Analysis

IBM SPSS version 22 software (IBM Corporation, Armonk, NY, USA) was used in the data analysis. Chi-square and ordinal regression procedures were used to determine the factors associated with levels of anxiety. All the variables that were significant at 0.25 level in the chi-square tests were tested in multivariate ordinal regression analysis.

### 2.4. Ethical Approval

This study has obtained ethical consent from the institutional review board (IRB) of the university (PSU IRB-2020-04-0038).

## 3. Results

### 3.1. Demographic Analysis

In this study, a total of 1054 responses were received. After data cleaning, 983 responses were found to be usable. The demographic characteristics of the respondents are shown in Table 1. Among the 983 respondents, 66.4% were females, majority (85%) were in the age group of 19–25 years, and almost one-half were Malays. Proportions of students from public and private universities were similar. In terms of field of study, about 95% were enrolled in either management studies, sciences, or health sciences. Almost three-quarters of the respondents were pursuing their undergraduate studies, and about 70% of them were either in their first or second year of study. At the time of data collection, almost all the universities were on virtual mode of delivery. Most (86.5%) of the students in this study stayed in their family homes with their family members.

### 3.2. Levels of Anxiety among University Students during the Pandemic

Internal consistency of the 20 items in the self-rating anxiety scale (SAS) was high (Cronbach’s alpha = 0.944). Based on Zung’s Anxiety Index, out of the 983 respondents in the sample, 201 (20.4%), 65 (6.6%), and 28 (2.8%) experienced minimal to moderate, marked to severe, and most extreme levels of anxiety, respectively (Table 2). Due to the low frequencies, cases with marked to severe anxiety and most extreme anxiety were grouped together and named as “Severe to Extreme” level of anxiety in further analysis.

### 3.3. Factors Associated with University Students’ Anxiety Levels during the Pandemic

#### 3.3.1. Univariate Analysis

The results from the chi-square analyses for the tests of associations between students’ demographic variables and anxiety are presented in Table 3. Among the tested variables, gender, age, ethnicity, type of university, field of study, and living arrangement were significant at a 0.25 level.

#### 3.3.2. Ordinal Regression Analysis

Gender, age, ethnicity, type of university, field of study, and living arrangement were tested in the ordinal multivariate regression analysis. In the analysis, the model fit was acceptable (deviation chi-square = 598.102, df = 643, *p* = 0.897). The *p*-value in the test of parallel lines was 0.117. Hence the equal-proportion assumption was met. As shown in Table 4, female gender (OR = 21.456, 95% CI = 1.061, 1.998, *p* = 0.020), age below 18 years (OR = 4.147, 95% CI = 1.331, 12.918, *p* = 0.014), age 19 to 25 (OR = 3.398, 95% CI = 1.431, 8.066, *p* = 0.006), pre-university level of education (OR = 2.882, 95% CI = 1.212, 6.854, *p* = 0.017), management studies (OR = 2.278, 95% CI = 1.526, 3.399, *p* < 0.001), and staying alone (OR = 2.208, 95% CI = 1.127, 4.325, *p* = 0.021) were significantly associated with higher levels of anxiety.

## 4. Discussion

This study examined anxiety among university students in Malaysia during the COVID-19 pandemic and the lockdown period between April and May 2020. Based on the findings, 20.4%, 6.6%, and 2.8% of the students experienced minimal to moderate, marked to severe, and most extreme anxiety levels, respectively. The results in this study are similar to that of [11,12,33].

The odds of anxiety were higher among the female students compared to that among the male students. This finding is similar to that of [49,50]. Females generally express emotions to a greater extent than males do, and the recent pandemic may have exacerbated this situation. Studies indicate that females’ uncertainty tolerance threshold is lower than that of males, and crossing that threshold triggers undue stress and anxiety. Female students may further be subject to lesser coping strategies in times of uncertainty and stressful situations.

In this study, the younger students, specifically those in the age group of 17 to 18 years, were more anxious compared to the older ones. As widely known, the youngsters are constantly on social media and the information shared on social media could have played a pivotal role in increasing the anxiety level of the students [46]. Although social media gives easy access to information, which can be essential during the lockdowns, the “always-on” facet of social media can be exhausting and may take a toll on students’ mental health. The flow of risk-elevating messages on social media that are portrayed in a very negative manner could trigger anxiety; 24/7 media coverage may make it seem like COVID-19 is omnipresent as well.

With regard to the field of study, students in management-related studies seemed to have a higher level of anxiety compared to healthcare and medical students. This is contrary to another study [51], which conjectured that medical and healthcare students experience a higher level of anxiety during times of epidemic or pandemics. The exact reason for the difference is not firm, but there is a possibility that the healthcare students could have been well-informed on what to expect as the pandemic progresses compared to the students of business- or management-related studies.

Students who were staying alone experienced the highest anxiety levels compared to those staying with family and friends. As it is, those staying alone are usually away from their loved ones and the sudden threat to their safety and security during this pandemic could have made these students feel lonelier and posed challenges from multiple angles. Tracking prolonged loneliness and swift interventions are imperative in reducing feelings of anxiety as they endorse a sense of belonging. Building and maintaining relationships is pivotal for mental and social well-being and is one of the hallmarks of student life. Unfortunately, the COVID-19 pandemic has created a “social recession”—a continual pattern of social distancing, beyond the immediate pandemic, that is creating a lack of emotional support and broader societal effects, which include increased anxiety levels.

Based on the selected relevant narrative feedback given by the students in the survey (Appendix A, Table A1), the most commonly highlighted stressors were predominantly financial constraints, remote online classes, and uncertainty about the future due to COVID-19 and lockdowns. In terms of finances, the students were concerned with their ability to manage their educational financial commitments due to family loss of income and loss of opportunities to work and self-finance their studies. Second, an important contributor to anxiety and stress level was the sudden move to online classes, better known as ERT. The students faced uphill tasks in terms of technological infrastructure, mainly poor internet connection. It is also appalling to note that some students attended 6–8 h of daily online classes using their mobile phones, which further contributed to insurmountable stress and health issues. In addition, the overwhelming expectations from their instructors, with multiple assignments and no flexible deadlines, added to the students’ anxiety. Uncertainty regarding their exams, completion of their semester and graduation, and the need to juggle household chores and take care of siblings while concurrently attending online classes had a huge impact on their anxiety levels. Students, especially those graduating, were also distressed because they were helpless in their plans to launch their careers.

Moreover, although universities promptly implemented remote online classes, most instructors still used the same curricula and learning outcomes meant for face-to-face teaching. This did not augur well with students and added tremendous stress and anxiety as they were excessively burdened with continuous alternative assessments. Many instructors fail to realize that the students are wading through complicated emotions due to COVID-19 and lockdowns and the fact that they have to adjust to remote learning and being isolated from their friends creates undue frustration, anger, resentment and ultimately, anxiety.

Further research is proposed for the inclusion of successful coping strategies used by the students during testing times such as the COVID-19 pandemic. Research should also be channeled toward teaching, learning, and assessment methods in the “new normal” space, which can have the dual benefit of maximizing learning outcomes and minimizing anxiety and adverse psychological impacts among students. Lastly, research focusing on the use of digital technology and psychological artificial intelligence solutions to manage anxiety levels of university students should be intensified.

## 5. Conclusions

The empirical evidence from this study indicates that 20.4%, 6.6%, and 2.8% of the students experienced minimal to moderate, marked to severe, and most extreme anxiety levels, respectively during the COVID-19 pandemic and lockdown period. Age, gender, academic specialization, and living condition were significantly associated with anxiety levels. Stressors were predominantly financial constraints, remote online learning, and uncertainty related to their academic performance, graduation, and future career prospects. To mitigate anxiety levels amongst students, the Centers for Disease Control (CDC), has proposed some guidelines—minimize repeatedly watching, reading, and listening to new stories on COVID-19, specifically from unreliable sources of social media; maintain healthy diet, exercise, adequate hours of sleep; and keep the mind stress free by virtually connecting with friends and family members; and finally, take time to unwind and indulge in activities one enjoys. As suggested by [52], it helps to feel that “everyone is in this together.”

There is a strong call for all stakeholders in the education industry to recognize the need for an immediate and holistic policy to identify and manage the psychological impact of COVID-19 or any future pandemics on students. In this regard, both higher education institutions and the relevant ministries at a broader level play a pivotal role.

Higher education institutions can play a fundamental role in assisting students to cope with such anxieties. New guidelines for counseling are mandatory [13,17,46]. Universities should set priorities in developing digital psychological interventions, such as apps and online programs, alongside other services such as text messages, chatlines, forums, and phone calls [53]. Awareness of the presence of such interventions should be clearly communicated to the student population. Universities should also provide psychological services, either face to face or remotely, as they will mitigate the emotional and mental impacts on students. It is crucial to be constantly in touch with the students. Universities ought to embark on structured programs to reduce anxiety, such as life skills training and mindfulness therapy, which have been validated to reduce anxiety levels. Equally important is for universities to re-examine their curricula, learning outcomes, and assessment methods for the courses and programs taught online as they should be distinguished from those meant for face-to-face teaching mode.

From a broader perspective, ministries and related agencies in coordination with the WHO, UNESCO, and CDC need to intensify community mindfulness, specifically for the students, by using artificial intelligence to obtain evidenced-based and scientific measures for pandemics. Most importantly, an all-inclusive teaching and learning strategy during pandemics should be deliberated immediately, as this study confirms that the emergency remote teaching has contributed to significant anxiety among students. Policies and standard operating procedures (SOPs) should be in place to educate students on the causes and consequences of pandemics in a simplified, clear, and supportive manner without causing undue anxiety and distress. Communicating correct and timely information through the right channels is important. Efforts should also be focused toward discovering innovative methods of upholding social attachment amongst students while still complying with public health guidelines for curtailing the spread of the pandemic. Strict measures and penalties should be enforced against unscrupulous individuals to curb false information via social media, as this seems to be a chief source of undue anxiety amongst students.

As in all surveys using questionnaires, there is always uncertainty of whether the respondents answered the questions honestly. In this study, data were collected in the months of April and May, 2020. By this time, most students had already moved back to their family homes as the university residences were closed. The anxiety levels of the students could have been different if the data had been collected at the peak period of COVID-19, which was in mid-March 2020. This, however, was not possible as we had to wait for the ethical approval from the relevant authorities.

## Figures and Tables

**Table 1:** Demographic characteristics of the respondents.

Variable	Frequency	Percentage
**Gender**		
Female	653	66.4
Male	330	33.6
**Age**		
17–18 years	37	3.8
19–25 years	836	85.0
Above 25 years	110	11.2
**Ethnicity**		
Malay	456	46.4
Chinese	215	21.9
Indian	270	27.5
East Malaysian	42	4.3
**University**		
Public	497	50.6
Private	486	49.4
**Field of study**		
Pre-University	38	3.9
Management Studies	295	30.0
Sciences	259	26.3
Arts/Communication/Languages	118	12.0
Health Sciences	273	27.8
**Level of Study**		
Pre-University	54	5.5
Diploma/certificate	108	11,0
Degree	714	72.6
Postgraduate	107	10.9
**Year of study**		
Year 1	359	36.5
Year 2	311	31.6
Year 3	225	22.9
Year 4	47	4.8
Year 5	41	4.2
**Virtual learning**		
Yes	927	97.2
No	27	2.8
**Current accommodation**		
Outside campus	54	5.5
Residency	79	8.0
Family home	850	86.5
**Currently staying with**		
Alone	42	4.3
Friends	83	8.4
Family	858	87.3

**Table 2:** Anxiety level based on Zung’s classification.

Anxiety	Frequency	Percentage
Normal	904	92.0%
Mild to moderate anxiety	51	5.2%
Moderate to severe	28	2.8% *

* Comprises both marked to severe anxiety: 23 (2.3%) and most extreme anxiety: 5 (0.5%).

**Table 3:** Results from univariate analysis.

Variable	Normal	Mild to Moderate Anxiety	Moderate to Severe	Chi Square	*p*-Value
**Gender**				7.270	0.026
Female	590 (90.4%)	42 (6.4%)	21 (3.2%)		
Male	314 (95.2%)	9 (2.7%)	7 (2.1%)		
**Age**				8.709	0.069
Below 18 years	32 (86.5%)	5 (5.4%)	3 (8.1%)		
19–25 years	765 (91.5%)	46 (5.5%)	25 (3.0%)		
Above 25 years	107 (96.4%)	4 (3.6%)	0 (0%)		
**Ethnicity**				10.643	0.100
Malay	407 (89.3%)	32 (7.0%)	17 (3.7%)		
Chinese	203 (94.4%)	8 (3.7%)	4 (1.9%)		
Indian	255 (94.4%)	6 (3.0%)	7 (2.6%)		
East Malaysian	39 (92.9%)	3 (7.1%)	0 (0%)		
**Type of University**				4.450	0.108
Public	449 (90.3%)	29 (5.8%)	19 (3.8%)		
Private	455 (93.6%)	22 (4.5%)	9 (1.9%)		
**Field of Study**				11.581	0.171
Pre-University	33 (86.8%)	4 (10.5%)	1 (2.6%)		
Management Studies	264 (89.5%)	19 (6.4%)	12 (4.1%)		
Sciences	236 (91.1%)	13 (5.0%)	10 (3.9%)		
Arts/Communication/Languages	113 (95.6%)	3 (2.5%)	2 (1.7%)		
Health sciences	258 (94.5%)	12 (4.4%)	3 (1.1%)		
**Level of Study**				4.264	0.641
Pre-University	49 (90.7%)	4 (7.4%)	1 (1.9%)		
Diploma/Certificate	102 (94.4%)	3 (2.8%)	3 (2.8%)		
Degree	651 (91.2%)	41 (5.7%)	22 (3.1%)		
Postgraduate	102 (95.3%)	3 (2.8%)	2 (1.9%)		
**Year of Study**				4.089	0.848
Year 1	336 (93.6%)	14 (3.9%)	9 (2.5%)		
Year 2	283 (91.0%)	18 (5.8%)	10 (3.2%)		
Year 3	205 (91.1%)	15 (6.7%)	5 (2.2%)		
Year 4	43 (91.5%)	2 (4.3%)	2 (4.3%)		
Year 5	37 (90.2%)	2 (4.9%)	2 (4.9%)		
**Virtual Education**				2.440	0.295
Yes	850 (91.7%)	51 (5.5%)	26 (2.8%)		
No	27 (100%)	0	0		
**Accommodation**				0.712	0.950
Outside campus	51 (94.4%)	2 (3.7%)	1 (1.9%)		
Residency	72 (91.1%)	5 (6.3%)	2 (2.5%)		
Family home	781 (91.9%)	44 (5.2%)	25 (2.9%)		
**Living Arrangement**				7.253	0.123
Alone	35 (83.3%)	4 (9.5%)	3 (7.1%)		
Friends	78 (94.0%)	5 (6.0%)	0 (0%)		
Family	791 (92.2%)	21 (5.2%)	28 (2.8%)		

**Table 4:** Results from ordinal multivariate analysis.

Parameter	B	SE	*p*-Value	OR (95% CI)
**Gender**				
Female	0.816	0.303	0.007	2.261 (1.248, 4.100)
Male	*ref*			1
**Age**				
Below 18 years	1.965	0.809	0.015	7.138 (1.461, 34.879)
19–35 years	1.357	0.618	0.028	3.884 (1.156, 13.046)
Above 35 years	*ref*			1
**Ethnicity**				
Malay	0.563	0.638	0.378	1.755 (0.502, 6.133)
Chinese	0.012	0.721	0.987	1.012 (0.247, 4.151)
Indian	0.109	0.700	0.876	1.116 (0.283, 4.399)
East Malaysian	*ref*			1
**Type of University**				
Public	0.475	0.299	0.113	1.607 (0.894, 2.889)
Private	*ref*			1
**Field of Study**				
Pre-University	1.237	0.612	0.043	3.446 (1.039, 11.433)
Management studies	0.993	0.351	0.005	2.699 (1.356, 5.371)
Sciences	0.696	0.366	0.057	2.005 (0.979, 4.106)
Arts/Communication/Language	−0.092	0.548	0.866	0.912 (0.312, 2.669)
Health sciences	*ref*			1
**Staying Arrangement**				
Alone	1.349	0.4664	0.004	3.852 (1.546, 9.599)
Friends	−0.202	0.504	0.669	0.817 (0.304, 2.196)
Family	*ref*			1

## Appendix A

**Table A1:** Students’ qualitative feedback.

“I’m struggling a lot by being at home because not everyone is blessed with a stable and happy family. I need to learn how to cope with it.” “Online class is more tired than the actual one. I barely can sleep at night and doing the assignments. The students need to use laptop early in the morning until midnight. This one of the reasons why the students not energetic.” “As in financial problem, I just do not want to be a burden to my family as my expenses are quite high.” “Uncertain of the upcoming academic plans and the coping abilities of students in academics after MCO lifted.” “In this current situation, they are a lot of things that make me worried such as can I finish my semester? Do I need to extend my study? How about my final exam?” “Cannot catch-up with online study.” “Couldn’t study or concentrate when having online class because need to do housework on time and the feedback from lecturer is too late. It’s really difficult to cope up with 2 subjects this semester and I have no idea with next long semester.” “Online classes—Struggling not to hang myself or jump off the apartment.” “Home is in Pahang and campus is in KL, although online class is implemented but still renting dorms/units is still one of my concerns because online classes might change back into normal class.” “I think online distance learning rise my level of stress.” “I hope that lecturers not “bully” students by giving A TON OF ASSIGNMENT WITH SHORT DUE DATE. It is not easy as they think. Sometimes I felt so stressed due to the short due date and the assignments. We are human too. I hope by signing this “petition” can help students. Please ” “Financial burden is already bad. No support system for students. Lecturers need to know the burden of students. Uncertainty of semester, research, etc.”
